# Two Decades of Advances and Limitations in Organ Recellularization

**DOI:** 10.3390/cimb46080543

**Published:** 2024-08-22

**Authors:** Alina Stoian, Aisha Adil, Felor Biniazan, Siba Haykal

**Affiliations:** 1 Latner Thoracic Research Laboratories, Division of Thoracic Surgery, Toronto General Hospital Research Institute, University Health Network, Toronto, ON M5G 1L7, Canadafelor.biniazan@uhn.ca (F.B.); 2 Department of Laboratory Medicine and Pathobiology, Temerty Faculty of Medicine, University of Toronto, Toronto, ON M5S 1A8, Canada; 3 Translational Biology and Engineering Program, Ted Rogers Centre for Heart Research, Toronto, ON M5G 1M1, Canada; 4 Reconstructive Oncology, Division of Plastic and Reconstructive Surgery, Smilow Cancer Hospital, Yale, New Haven, CT 06519, USA

**Keywords:** decellularization, recellularization, organ recellularization, extracellular matrix

## Abstract

The recellularization of tissues after decellularization is a relatively new technology in the field of tissue engineering (TE). Decellularization involves removing cells from a tissue or organ, leaving only the extracellular matrix (ECM). This can then be recellularized with new cells to create functional tissues or organs. The first significant mention of recellularization in decellularized tissues can be traced to research conducted in the early 2000s. One of the landmark studies in this field was published in 2008 by Ott, where researchers demonstrated the recellularization of a decellularized rat heart with cardiac cells, resulting in a functional organ capable of contraction. Since then, other important studies have been published. These studies paved the way for the widespread application of recellularization in TE, demonstrating the potential of decellularized ECM to serve as a scaffold for regenerating functional tissues. Thus, although the concept of recellularization was initially explored in previous decades, these studies from the 2000s marked a major turning point in the development and practical application of the technology for the recellularization of decellularized tissues. The article reviews the historical advances and limitations in organ recellularization in TE over the last two decades.

## 1. Introduction

When discussing treatment options available for patients suffering from end-stage organ failure, transplantation is the only option. However, because of organ scarcity, only a small number of these patients benefit from transplantation [1]. Tissue bioengineering aims to solve donation shortages and immune rejection by regaining or restoring the damaged or lost function of organs by fabricating tissue-engineered organs [2,3]. Several strategies are being explored to achieve this goal, but traditional TE is based on the interplay between cells and biocompatible scaffolds, and the technique of whole organ decellularization and recellularization has attracted increasing attention in the last two decades [1,2]. The recent introduction of acellular organ scaffolds (AOS) in the field of TE has facilitated progress toward whole organ fabrication [4]. The concept of recellularization was initially explored in previous decades, and the 2000s studies marked a major turning point in the development and practical application of the technology for the recellularization of decellularized tissues. This next frontier of recellularization is an evolving area of TE, where the goal is generating recellularized organs at the scale of human size for transplantation. Recellularization is a dynamic process of repopulating acellular organ scaffolds post-decellularization with patient-specific cells, using either in vivo or ex vivo environments (e.g., bioreactors). Ultimately, the ideal is to use autologous cell sources (e.g., stem cells) for recellularization to develop patient-specific organs for transplantation; however, another potential option is genetically engineering immune-compatible cells [5].

Therefore, organ recellularization points to a few exciting avenues. This method can help circumvent or minimize the need for immunosuppression regimens post-transplantation. The retained ECM post-decellularization can ideally serve as a microenvironment for recellularized cells to engraft, proliferate, and function. The retained vasculature in decellularized scaffolds can also help facilitate nutrient and oxygen exchange post-recellularization [6]. There has yet to be a standardized recellularization approach for organs, because much of the work thus far has relied on a “black box” approach. Many of the studies have employed various techniques to achieve organ recellularization across small and large animal models, as well as human cadaveric organs. In assessing these studies and the progress in organ recellularization thus far, the principles and techniques required to achieve recellularization of organs are therefore critical to discuss. Once a decellularized scaffold is developed, there are multiple essential factors for achieving effective recellularization, such as recapitulating the native vasculature, obtaining and delivering multiple organ-specific cell types to respective locations within organs (e.g., vascular, parenchymal), testing and employing multiple seeding strategies for enhanced cell coverage, and deriving physiologically relevant bioreactors to culture and recellularize organs [7,8]. The recellularization of acellular organs also consequently requires the restoration of physiological function, which considerably depends on various cell types, compositions, cell–cell and cell–matrix interactions, and functions. Yet, while mono- and co-culture approaches have been taken with organ recellularization, there has not been a successful recellularization of organs to a complex state that contains multiple types of cells, such as vascular, parenchymal, and stromal cells altogether, which would significantly progress organ recellularization further [8]. In discussing these factors, maintaining a perfusable vascular tree is key to ensuring the delivery of oxygen and nutrients, as well as an ability to integrate and maintain scaffolds after in vivo implantations [7,9,10]. Achieving full revascularization is an essential prerequisite for in vivo and clinical applications given the risk of thrombosis if vascular walls are bare [11]. With expected ECM structure maintenance post-decellularization, this preserved native inner vascular network serves as a conduit to reach parenchymal and void spaces within organs that otherwise are difficult to reach and therefore difficult to repopulate. This would likely entail having repopulated cells migrate from the vascular network into the interstitial space as well as the parenchyma [8]. Therefore, while efforts have been made across various organs in recellularizing and repopulating the vasculature, another aspect to consider is maintaining adequate coverage post-recellularization. Recellularization strategies also rely on and must be tailored according to organ-specific functions. When evaluating recellularized organs, each type of organ requires tailored and different tests to determine the functional capacity post-recellularization. For example, recellularized hearts require showing the pumping of blood, recellularized lungs would require cells to be able to exchange oxygen and carbon dioxide, whereas recellularized kidneys should show fluid balance maintenance via filtration, reabsorption, and developing urine [11,12]. Therefore, cell seeding strategies have been organ-specific and have employed both vascular and non-vascular routes to achieve better cell coverage [6,7].

This article reviews the historical advances and limitations in organ recellularization, emphasizing all important aspects of this process. A summary of key organ recellularization studies is also provided in this paper.

## 2. Decellularization

The concept of decellularization, which involves removing cellular components from tissues or organs while preserving the extracellular matrix, has been described and developed over several decades. The goal of organ decellularization is the removal of all cellular material without affecting the composition, biologic activity, or mechanical integrity of the remaining three-dimensional (3D) matrix [13].

Optimizing the decellularization step is crucial for the creation of a naturally derived, well preserved 3D ECM that provides the functional support needed for cell growth [14]. It is believed that the application of scaffolding materials can achieve enormous potential for tissue regeneration. Despite the widespread application of several tissue-engineered scaffolds (Table 1), some of them have limited repair capacity [15].

**Table 1:** Types of scaffolds used in TE.

Type of Scaffold			
Synthetic Scaffolds		Natural Scaffolds	
Hybrid Scaffolds			
Based on Degradable Polymer	Based on Non-Degradable Polymer	Based on Degradable Polymer	Decellularized Extracellular Matrix
Poly(lactic acid) Poly(glycolic acid) Poly(ε-Caprolactone) Poly(glycerol sebacate) etc.	Polyurethane Synthetic elastin Polyethylene terephthalate Expanded polytetrafluoroethylene etc.	Collagen Elastin Fibrin Chitosan/CS Silk fibroin etc.	Obtained by decellularization of organs/tissues
[16,17,18,19,20,21,22,23,24,25,26,27,28,29]			

Exploring a gold-standard scaffold—decellularized ECM (dECM) is a type of scaffold that mainly consists of extracellular matrix (ECM), which is a 3D structure framework containing extracellular macromolecules (collagen, elastin, fibronectin, and laminin) and matricellular proteins [30]. The physicochemical signals and biological performance of ECM can be maintained after decellularization and provides, in this way, a substrate for mechanical support and a biological 3D carrier for subsequent cell seeding. The consequent cell-seeded construct based on dECM scaffolds is considered an ideal choice for regenerating functional organs/tissues [9,15,31]. Decellularization is now a basic method in obtaining dECM scaffolds. Decellularization is a process that removes cells from organs or tissues, resulting in a cell-free scaffold consisting of the tissues’ extracellular matrix [2]. Crapo et al. listed, in 2011, the following minimal suffice criteria of decellularization: (i) <50 ng dsDNA per mg ECM dry weight, (ii) <200 bp DNA fragment length, (iii) lack of visible nuclear material in tissue sections stained with 4′,6-diamidino-2-phenylindole (DAPI) or H&E [32]. Several decellularization techniques have been developed to date to reconstruct different types of organs/tissues. Generally, decellularization methods are classified into three main groups: chemical methods, physical methods, and biological methods, with specific advantages and disadvantages for each group (Table 2) [33].

**Table 2:** Decellularization techniques used in TE.

Type	Advantages	Limitations
**Physical methods**		
- Freeze-thaw cycles - Agitation - Pressure - Supercritical fluids - Nonthermal irreversible electroporation (NTIRE) - Ultrasound - Perfusion decellularization	- Can be gentler on the ECM compared to chemical methods - Can be precisely controlled and tailored to the specific tissue type - Less concerns about residual chemicals that may affect biocompatibility of the ECM [32,34,35]	- May require combination with chemical or enzymatic methods for complete decellularization - Some methods require specialized equipment - Effectiveness can vary depending on tissue type and density - May disrupt ECM components over prolonged exposure [32,36,37,38]
**Chemical methods**		
- Acids and bases - Ionic, non-ionic, and bipolar detergents - Solutions - Chelating agents - Alcohols	- Completely removes cellular components - Applicable to all organs and tissue types - Customizable protocols for specific tissues [32,36,39,40]	- ECM damage (harsh chemicals can degrade ECM) - Residual chemicals (potential cytotoxicity and immune response) [38,41,42]
**Biological methods**		
- Esterase - Protease - Nuclease	- Expedite the removal of DNAs and RNAs from the scaffold - Enzymes used in biological methods are typically biocompatible and less cytotoxic compared to harsh chemical agents [33,43]	- Cleaving ester bonds in the cell membrane may disrupt ECM components - Variability in enzyme concentration, pH, and temperature can affect decellularization outcomes and require meticulous optimization and monitoring [33,34,44]

However, even though most of the studies claim preservation of the ECM ultrastructure after decellularization, almost all methods used for this aim would inevitably cause a certain degree of disruption to the ECM structure, and some studies question, “How good is good enough?” [45]. Therefore, it is essential to preserve most of the native ECM during the decellularization; these tissues can be utilized for tissue remodeling and regeneration as grafts, provided the ECM remains functional and offers the necessary signals to support cellular growth and differentiation, guiding the formation of new tissue [46]. dECM properties play a crucial role in organ recellularization by regulating cell functions and promoting the formation of new tissues and organs [47].

## 3. Recellularization—A Dependent Process

### 3.1. dMEC—“How Good Is Good Enough?”

The ECM represents the secreted products of the resident cells of each tissue and organ, and it is in a dynamic reciprocity with these cells in response to changes in the microenvironment and has been shown to provide cues that affect cell migration, proliferation, and differentiation [32]. The interaction between cells and the ECM is both dynamic and reciprocal. Cells continuously receive environmental information from ECM cues and are actively involved in remodeling their surrounding ECM [48]. However, the ECM is not only essential as a physical scaffold for cellular constituents, but it also initiates crucial biochemical and biomechanical signals for tissue morphogenesis, differentiation, and homeostasis. The ECM is a highly dynamic structure that undergoes continuous remodeling, with its molecular components experiencing numerous post-translational modifications. These physical and biochemical characteristics of the ECM endow each organ with specific biochemical and mechanical properties, such as tensile and compressive strength, and elasticity. Additionally, the ECM provides protective functions by buffering to maintain extracellular homeostasis and water retention. Furthermore, the ECM guides crucial morphological organization and physiological functions by binding growth factors (GFs) and interacting with cell-surface receptors to initiate signal transduction and regulate gene transcription. The biochemical, biomechanical, protective, and organizational properties of the ECM can vary significantly between different tissues [49].

The ECM is mostly a protein-based (over 300 types) structure that acts as a skeleton to support and hold cells [50]. The ECM is also a reservoir of growth factors and bioactive molecules. It is a highly dynamic structure that has vital importance for the fundamental behaviors and characteristics of cells in the tissue [51]. Collagen, elastin, and glycosaminoglycans (GAGs) are the most important structures in the ECM. Collagen and elastin play a critical role in controlling tissue osmotic pressure and regulating intracellular signaling cascades for cell differentiation and function, while GAGs serve as crosslinkers for carrying GFs [52]. Regarding the most important features of the dECM, biocompatibility, biodegradability, and angiogenic potential are crucial properties of the dECM.

#### 3.1.1. Biocompatibility

Over time, various definitions of biocompatibility have emerged. The ideas about biocompatibility began to take shape around the same period that cytokines and cell surface receptors were identified. However, these groundbreaking discoveries were not incorporated into the initial definitions of biocompatibility [53]. The most widely used definitions for biocompatibility were offered by Bing J. in 1955—“Biocompatibility is the ability of a material to perform with an appropriate host response in a specific application” [54]—and by D.F. Williams in 1981—“Biocompatibility refers to the ability of a material to perform its desired function with respect to a medical therapy, without eliciting any undesirable local or systemic effects in the recipient or beneficiary of that therapy” [55]. According to Buddy D. Ratner, contemporary definitions of biocompatibility for biomaterials are linked to the presence of a thin, stable foreign body capsule and minimal cellular response at the site of the implant [53]. In tissue engineering, biocompatibility for dECM can be characterized by its lack of immunogenicity, minimal toxicity, and its ability to support cell attachment, spreading, and healthy proliferation through strong adhesion and supportive features [46,56,57].

#### 3.1.2. Biodegradability

The concept of biodegradability, especially in the context of materials and their applications in tissue engineering, has evolved over time. One of the early mentions of the importance of biodegradability in tissue engineering can be traced back to the work of researchers in the 1980s and 1990s who explored biocompatible and biodegradable materials for medical implants and scaffolds [58]. Langer and Vacanti discussed the necessity of using biodegradable materials for creating scaffolds that support tissue regeneration; they highlight how controlled degradation is crucial for allowing new tissue to grow and integrate properly, thus underscoring the importance of biodegradability in tissue engineering [59]. ECM remodeling is a tightly controlled complex process, with many proteins playing important roles to maintain homeostasis. The renewal rate of the ECM in organs varies depending on the type of tissue and its location in the body [60]. The biodegradation of the ECM happens through the process of remodeling. This involves the breakdown and removal of old or damaged ECM components by enzymes and other proteinases. These enzymes degrade the ECM, allowing for the turnover and renewal of the matrix, which is essential for normal cellular and organ function [61]. The balance between ECM synthesis and degradation ensures proper tissue maintenance and repair. Maintaining ECM homeostasis and remodeling is crucial for normal cellular and organ function, and this process is controlled by two families of enzymes. The primary enzymes involved in modifying the ECM are matrix metalloproteinases (MMPs), a superfamily of zinc-dependent endopeptidases. MMPs, along with other proteinases like plasmin, thrombin, and cathepsins, are produced as inactive zymogens that require proteolytic cleavage of a self-inhibitory pro-domain for activation. The activity of MMPs is regulated by specific inhibitors known as tissue inhibitors of metalloproteinases (TIMPs) [62,63]. For collagens and elastin, intermolecular cross-linking by enzyme lysyl oxidases (LOX) is a key post-translational modification. Expanded cross-linking due to excess LOX activity increases ECM stiffness, affecting cellular behaviors. The ECM is also modulated by exogenous stimuli (cytokines, glucocorticoids, oxidative stress, pressure, and mechanical stretch). Transforming growth factor-β (TGF-β) is the most studied cytokine and is known to enhance ECM production and upregulate ECM-related genes [51].

The angiogenic potential refers to the ability of the dECM to promote the formation of new blood vessels. This is crucial for tissue engineering and regenerative medicine, as the formation of new blood vessels ensures adequate blood supply, oxygen, and nutrients to the engineered tissue and organs [64]. The first vessel obtained through tissue engineering was conceived and designed in the mid-1980s by Weinberg and Bell, who used endothelial cells from bovine aorta, smooth muscle cells, and fibroblasts in a collagen ring matrix with smooth muscle cells. Although the authors created a vessel with weak mechanical properties at that time, they laid the foundation for the protocol still used today in TE to obtain functional vascular grafts. This protocol is based on three main elements: multipotent cells, an extracellular scaffold, and growth factors [65]. Endothelial cells interact with the ECM and offer the support for angiogenesis, and the key activator of endothelial cells is vascular endothelial growth factor (VEGF). These molecules are responsible for endothelial cell migration into normal tissue and initiating cell division and self-organization into vessels [66]. The angiogenic potential of dECM depends on its composition, structure, and the presence of bioactive molecules that can stimulate endothelial cells and other components involved in the angiogenic process. By controlling the physiological properties of the ECM, it is possible to modulate the vascular network [67]. The number of ECM molecules that can influence angiogenesis is increasing; Mongiat et al. describe around 28 ECM molecules involved in angiogenesis [68]. The transplanted dECM not only provides a scaffold for new tissue growth but also actively participates in the biochemical signaling necessary for angiogenesis by releasing growth factors and interacting with the host’s cellular machinery [69].

Angiogenesis is a process involving the interplay between growth factor (GF) and integrin-mediated mechanosensing, leading to endothelial morphogenesis and the formation of new tubular structures [70,71]. This process begins in response to hypoxia, which triggers the release of pro-angiogenic GFs. These cell-secreted GFs diffuse through the interstitial space and bind to sulfated ECM molecules, which control their local biodistribution and receptor activation. Vascular endothelial GF-A (VEGF-A) is the primary modulator of angiogenesis. It binds to the VEGFR-2 receptor on endothelial cells (EC) and triggers the angiogenic cascade and vessel formation. VEGF-A exists in several isoforms, such as VEGF-A121, VEGF-A165, and VEGF-A189, each with different ECM binding affinities that influence vascular patterns. For example, VEGF-A121, which does not bind to the ECM, results in shorter, leakier vessels with larger diameters and fewer branches, commonly seen in tumors. VEGF-A189, with strong ECM binding, leads to aberrant branching and smaller capillaries. VEGF-A165, with intermediate ECM affinity, forms physiologically normal vascular structures, demonstrating that the strength of GF-ECM binding is crucial in determining angiogenic outcomes [72].

However, creating vascular networks in vivo based on dECM remains challenging, especially when it comes to recapitulating the complex anatomy and functionality of microvascular organotypic structures [73]. Only a limited number of engineered tissues, such as skin, cartilage, and bladder, have reached clinical success, while biomaterials intended to replace more complex organs remain commercially unavailable [74].

### 3.2. dECM Recellularization’s Limitations

Despite the numerous advantages of the dECM, its recellularization process presents a difficulty, especially due to some of the specific limitations [14].

Inhomogeneous cell distribution—one of the major milestones in recellularization procedures, and it refers to the non-uniform spread of cells. This can be a critical issue in medical applications, impacting the functionality and effectiveness of the tissue-engineered organ [75]. Cell migration within 3D tissue is influenced by a balance between the deformability of the cells and the physical constraints of the tissue. The cells’ migration is regulated by the capacity to degrade ECM by proteolytic enzymes (matrix metalloproteinases—MMPs) as well as by integrin- and actomyosin-mediated mechanocoupling [76]. There are several discussed methodologies, principles, and applications related to cell density calculations and strategies in tissue engineering. Schulze-Tanzil et al. described the inhomogeneous distribution of cells and cell-free areas in reseeded tendon ECMs in comparison to native tendons. The authors also describe how the seeded cells remain mostly inhomogeneously distributed, and they usually do not align into rows [75]. Rahvar and Abdekhodaie employed a continuum reaction-diffusion model to examine how encapsulation parameters affect cell response on microgels. The findings show that the inhomogeneous distribution of ECM is due to nutrient gradients, and microgels with radii under 200 μm achieve nearly uniform ECM deposition. The study also found that initial cell density does not significantly impact ECM distribution in these small microgels [77].

Cell adhesion and retention—apart from blood cells, most, if not all, other normal cells in the tissues are anchorage-dependent, residing in the ECM [78]. TE and regenerative medicine depend on gaining a deeper understanding of the interactions between cells and ECM for establishing and maintaining cell environments and subsequent tissue/organ homeostasis [79]. The organelles mediating cell–ECM interactions can be classified into two main categories: (i) focal adhesions and (ii) hemidesmosomes. Hemidesmosomes, which associate with intermediate filaments, are primarily found in epithelial tissues linked to a basement membrane. Focal adhesions, associated with actin filaments, are present in most adhesive cells and are major sites for cell–ECM interactions. Focal adhesions are highly dynamic organelles composed of structural and signaling components, including transmembrane integrins and actin regulatory proteins like α-actinin. They transmit cytoskeletal force generated by actomyosin contraction to the surrounding environment and can respond to external factors such as stiffness, tension, viscoelasticity, and stress relaxation. Focal adhesions are also crucial in regulating cell migration rates [79,80]. Adhesion receptors are typically clustered in cell junctions. Desmosomes and adherens junctions mediate cell–cell adhesion, while hemidesmosomes and focal contacts mediate cell–matrix adhesion. Intercellular junctions include tight junctions, which regulate paracellular permeability across the epithelium and determine cell polarity, and gap junctions, which facilitate intercellular communication. Besides their role in cell adhesion, adhesion receptors also transduce signals that regulate important aspects of cell behavior, such as movement, proliferation, differentiation, and survival. Cell adhesion is a dynamic and complex process, dependent on dECM composition, mechanical properties, biochemical signals, cell-specific requirements, and the presence of inhibitory molecules [81].

Mechanical integrity and stability—scaffolds provide mechanical and shape stability for tissue/organs, and the intrinsic mechanical properties of the scaffold should match that of the host tissue [78]. Cell–matrix interactions encompass not only the ECM’s chemical composition and structural organization but also its mechanical properties [82]. The mechanical properties of the dECM depend on components like elastic fibers, fibrillar collagens, GAGs, and the related proteoglycans (PGs) [82,83]. The studies on mechanobiology have highlighted the importance of mechanical properties of a dECM on the seeded cells. The feedback of local matrix stiffness on cell state likely has important implications for development, differentiation, disease, and regeneration. Exerting traction forces on a substrate, many cell types sense the stiffness of the substrate and show dissimilar morphology and adhesive characteristics [78,84].

Immunogenicity—it is believed that the absence of cells reduces the risk of immune responses, making the use of allo- and xeno-dECMs a viable approach [85]. Decellularization removes immunogen cell materials and significantly alleviates the immunogenicity and biocompatibility of dECM. However, the clinical application of these bioscaffolds still faces major immunologic challenges [86]. The interaction of dECM scaffolds with the immune system is primarily mediated by damage-associated molecular patterns (DAMPs), which are danger signals released during cell and ECM damage from graft processing or implantation. Notable DAMPs include nuclear and mitochondrial DNA, reactive oxygen species (ROS), and fragmented ECM components like hyaluronic acid or fibronectin. These DAMPs are released due to disruptions in cellular nucleic acid organization, transplantation issues such as ischemia-reperfusion, and aggressive decellularization techniques [45,86,87]. Technological advances in immunolabeling, cell sorting, genomics, proteomics, and other laboratory techniques have revealed the essential roles of the immune system in tissue and organ development. Regardless of the approach used in TE, the objective remains the same: the restoration of structure and function. The response of the host to the implanted construct will ultimately dictate the success or failure of the outcome [88,89]. Genetically engineered hypoimmunogenic cells are poised to become a standard approach in allogeneic cell therapy, potentially reducing or eliminating the need for immunosuppression in patients [90]. Until then, immunosuppressive protocols are a crucial component of transplantation experiments in nearly all experimental treatment strategies involving small or large animal models before clinical translation [91].

Cells selection—the creation of dECM for use as natural scaffolds involves efficiently decellularizing donor tissue, which can then be re-seeded with different types of cells to achieve functional outcomes [46]. Different cell types have been investigated for the repopulation of dECM to create functional organs, such as embryonic stem cells, adult stem cells, induced pluripotent stem cells (iPSC), perinatal stem cells, umbilical cord blood stem cells, cord tissue stem cells, dental pulp stem cells, and adipose-derived stem cells [64]. Ideally, candidate cells should be easily obtainable from patients, capable of being expanded in culture, and then reseeded into decellularized organ scaffolds where they exhibit tissue-specific differentiation [92]. iPSCs are among the most promising tools in regenerative medicine and TI today. Since their discovery by Shinya Yamanaka and his team in 2006 [93], numerous studies have highlighted their key characteristics of pluripotency, which enable them to differentiate into various cell types while retaining their capacity for multilineage differentiation [94,95].

### 3.3. dECM Recellularization’s Advances

While the effect of decellularization and maintenance of the ECM has been discussed as crucial for the success of recellularization above, the techniques used to conduct recellularization, such as developing bioreactor systems and seeding strategies, heavily influence the success and outcome of recellularization as well and warrant discussion.

#### 3.3.1. Bioreactors

Complex organs such as the heart, liver, lung, pancreas, and kidney have multiple requirements to ensure tissue formation and maturation during recellularization. Tissue thicknesses must also be accounted for, given the limited oxygen diffusion distance, ensuring that organs with higher tissue thickness (e.g., the heart) have a functional vascular supply of nutrients and oxygen to support cell survival and function during recellularization [96]. Therefore, ex vivo bioreactors can provide and are often designed to mimic physiologically relevant microenvironments to support cell survival and function in an organ-specific way [6]. Depending on organ size and the extent of recellularization, cell engraftment, growth, and maturation can take several weeks in a bioreactor setting [97]. To establish and maintain physiological conditions in ex vivo bioreactors, certain components are needed, such as, but not limited to, perfusion parameter monitoring (e.g., flow rate and perfusion pressure), pH/pCO2/pO2 level monitoring, metabolic parameters monitoring and maintenance (e.g., glucose, lactate, electrolytes), access ports for vascular and non-vascular routes of organs, bubble traps for air bubbles, and perfusion machines for either pulsatile or continuous perfusion for the duration of recellularization. These particular components are important to monitor real-time to ensure cell survival, differentiation, and maturation, especially during prolonged periods of culture. Additional components necessary to maintain recellularization long-term ex vivo include system design components such as preventing leaks, ensuring materials are sterilizable/autoclavable and easy-to-assemble, along with scalability, as the ability to withhold and perfuse large volumes of solutions is required for larger organs (e.g., pig or human cadaveric organs) relative to smaller organs (e.g., small animal models) [1,9,97].

Biophysical cues are also critical for tissue development during recellularization in various forms, such as chemical, mechanical, and/or electrical stimulation [9,97]. Organs such as the heart require electrical and mechanical stimulation, whereas organs such as the lung require ventilation of the lung to facilitate gas exchange [1]. For example, mechanical stretching of recellularized heart scaffolds helps promote the expression of certain cardiac markers (e.g., connexin-43) and influences cell alignment and function, whereas electrical stimulation can help develop contractile forces in recellularized cardiac cells [97,98,99]. Others, such as Visone et al., have developed cardiac patches in bioreactors with both electrical stimulation and bidirectional perfusion along with real-time monitoring of cells, resulting in an integrated biophysical environment that can show its effects real-time as the cardiac patches undergo maturation [100]. In terms of the lung, mimicking mechanical stimulation remains important during recellularization to ensure that, as lungs are inflated/deflated, the ECM can also maintain mechanical compliance [97]. Ventilators are often used to match the respiration patterns of either animal or human tissues the organs are derived from [101,102,103]. Other organs such as the kidney may incorporate chemical stimulation to facilitate recellularization [97].

There is also significant discourse on the next significant advancements needed in bioreactor development for organ recellularization. Non-invasive imaging and the monitoring of recellularized cell populations during ex vivo culture would represent a significant step forward in bioreactor development. This can include microcomputed tomography, near infrared imaging, laser doppler perfusion imaging, or multiphoton imaging, which represent potential avenues [9,97,104]. Other non-invasive options include using microparticles, as shown in recellularization of lungs, where microparticles were perfused in recellularized lungs to determine the extent of vascular patency [41,105].

To reach full clinical potential, these recellularized organs require both structural and functional capacities similar to native, healthy organs. Another factor in using ex vivo bioreactors is that they are single-organ systems that do not provide the complex interactions between tissues and different organ systems that normally occur in the human body. Given the scale of recellularization needed for human-sized organs, we may therefore have to rely on both ex vivo bioreactors and in vivo implantations to achieve full organ maturation. While our human bodies can be an ideal bioreactor, certain steps are required to ensure the integration of bioengineered organs in humans, such as the maturation of neo-organs up to their functional capacity, complete re-endothelialization of the vasculature to prevent thrombosis, and recellularized cells that have settled within their respective compartments and exhibit cellular growth and function [1].

#### 3.3.2. Seeding Strategies

Proper scaffold seeding, especially of the vascular system, is paramount to the survival of the bioengineered organ. While the use of bioreactors discussed above have a strong role in seeding strategies, scaffold seeding also relies on the delivery method, duration of seeding, culturing conditions, and cell number. Besides the vascular routes used to repopulate organs, non-vascular routes are also used for cell seeding (e.g., ureter for kidney). Further, the delivery method must also be carefully controlled to ensure that the biomechanical properties (e.g., pressure and shear stress) are also controlled in their influence on cell behavior [7]. There have been three predominant seeding strategies used for recellularization, including static, injection, and perfusion seeding. Static cell seeding methods have been employed for recellularization, such as through surface seeding; however, they result in superficial cell attachment [106]. Static seeding for organs also poses challenges due to a reduced cell number that can be statically seeded before reaching confluence at the surface [8]. Injection seeding, using a small gauge needle, allows direct and targeted delivery and has been trialed in heart and liver recellularization studies [40,107,108]. This method, however, has been shown to have low cell seeding efficiency in Ott et al.’s landmark rat heart recellularization study where cells were not well distributed post-injection [40]. Therefore, this seeding method may present limitations in migrating and positioning cells in distant vascular networks and parenchymal spaces. Alternatives include spinner flasks or a medium reservoir in which cells are added and cycled through a circuit into the organ. Such a strategy employed in recellularizing rat livers by Soto-gutierrez et al. resulted in low cell seeding efficiency [109]. Therefore, the recellularization of complex organs (e.g., kidneys) requires dynamic cell seeding to traverse the inner vasculature and achieve more coverage in the organ. Some studies have employed a combination of perfusion and injection seeding, such as in rat heart recellularizations [110]. Many studies commonly add highly concentrated cell suspensions through vascular perfusion and allow the cells to travel into the scaffold and parenchyma, either by static or perfusion culture, as seen in previous heart, liver, lung, pancreas, and kidney recellularization studies [7,40,109,111,112,113,114]. Perfusion seeding is advantageous due to a few reasons. It can facilitate cell delivery across larger distances and hard-to-reach areas within an organ. Preserving the ECM ultrastructure is therefore paramount in employing this seeding method to help reach niche areas. Further, some investigators have divided large cell suspensions into multiple successive inoculations to achieve better cell seeding coverage [109,112,114,115,116]. Uygun et al. and Soto-Gutierrez et al. reported superior seeding efficiency using this method in liver recellularization studies, showing better cell distribution across the organ [109,115]. Many similar studies using vascular routes for recellularization incorporate endothelial cells, sometimes in coculture with other stem cell or support cells, as a means for re-endothelialization. Examples include small-animal rat pancreas [112], rat heart [110], rat kidney [117], mouse lung [118], rat and human lungs [41,119], rat liver [120], and human liver [121,122]. Employing perfusion seeding with endothelial cells has shown the ability to develop an evenly distributed endothelium in liver and lung organ recellularization studies [119,120]. Improved cell function has also been observed in liver and lung recellularization with coculturing seeding methods [41,120]. While much work has been conducted, further work remains in improving re-endothelialization in organs in terms of culturing conditions, seeding duration, and delivery methods [8]. Many studies have taken additional measures to investigate coating agents and cross-linking agents to allow more systemic re-endothelialization [1]. Many of these studies have also largely relied on endothelial cells, either by progenitor populations or terminally differentiated cell lines. Some later studies, however, have included the use of induced pluripotent stem cells (iPSCs), such as with heart recellularization, with promising outcomes [40,123]. Further, the incomplete recellularization of parenchymal regions in organs remains a challenge and an ongoing area of further work [96]. Meanwhile, perfusion seeding via vascular networks has shown success, and non-vascular routes have also been employed with significant progress. Examples include using the ureter in the kidneys and the airway in the lungs [7].

## 4. Organ Recellularization

### 4.1. Heart

A fundamental organ of the circulatory system, the heart pumps blood in the body and orchestrates a balance between its electrical and mechanical functions. Ott et al. reported a pivotal rat heart decellularization study that helped broaden the scope of organ bioengineering using the technique of decellularization/recellularization [5,7,40]. Using various cell populations, such as neonatal cardiomyocytes and support cells (e.g., fibrocytes and smooth muscle cells), researchers performed injection seeding during retrograde perfusion in the left ventricle and re-endothelialization using rat aortic endothelial cells via infusion in the aorta. Coupled with electrical stimulation, the researchers found contractile responses in the bioengineered hearts. Subsequent work building on Ott et al.’s foundational study was employed in various animal models, as well as human cadaveric hearts, using multiple cell populations, including iPSCs. Examples of animal heart recellularization studies include those in the mouse [123], rat [124,125] porcine models [39,126,127]. Human heart de- and recellularization studies have also been conducted in the past decade with significant progress [128,129]. Nguyen and colleagues’ heart recellularization in the rat also stands out given their decellularization using both venous and arterial routes. Subsequent recellularization by both the superior vena cava and ascending aorta showed a wide dispersing of cells throughout the decellularized rat hearts. iPSC-derived cardiac cells also demonstrated repopulation in the recellularized rat heart after 3 weeks of culturing [125]. Multiple studies have employed injection-based seeding for heart recellularization [40,124,130]. Others have used the direct perfusion of cells using the vasculature and cultured them in bioreactors [123,131,132]. Studies by Robertson et al., Weymann et al., and Kitahara et al. have employed combination seeding strategies using both perfusion and injection seeding to recellularize rat and porcine hearts [110,133,134]. Notable progress has also been made since in the past decade, including Guyette et al.’s report of a partial recellularization of a human decellularized cadaveric heart. Researchers seeded and 2D-cultured sections of perfusion-decellularized human heart cardiac matrix with human iPSC-cardiac myocytes where spontaneous contraction was observed after 4–7 days until static culture. Whole heart recellularization was also performed with 500 million human iPSC-derived cardiomyocytes injected intramyocardially in the left ventricle and cultured in a human heart bioreactor for 14 days. Researchers showed metabolically active recellularized segments of the myocardium after 14 days, 90% cell viability, visible contractility upon electrical simulation, and spontaneous contractions [130]. Therefore, multiple studies have extended to large-scale recellularizations of porcine and human hearts to varying extents, even using iPSC-derived cardiomyocytes. Despite the progress, the heterotopic implantations in various small and large animal models have not shown considerable in vivo functionality that would help perfuse and maintain the bioengineered hearts long-term [30,40,110,133].

### 4.2. Lung

Lung recellularization studies have progressed significantly in the past two decades. In 2010, the first whole rat lung de- and recellularization studies were reported [135,136]. Routes of cell seeding included both the pulmonary artery and vein, using various cell populations. Ott and colleagues employed HUVECs, A549 cells, and primary fetal lung cells across periods of 5–9 days in their rat lung recellularization study. Their bioreactor featured a closed-system design with perfusion lines in the pulmonary artery, trachea, and left atrium. Mechanical stretch was incorporated in their bioreactor system similar to fetal breathing movements. The authors noted recellularization of the endothelium as well as the respiratory and alveolar epithelium [135]. Petersen and colleagues developed a negative-pressure ventilation-based bioreactor using a syringe-pump system. Recellularization was performed using both vascular and airway lung compartments for a period of 8 days using neonatal rat lung epithelial cells (airway) and microvascular lung endothelial cells (pulmonary artery). The authors reported vascular perfusion and showed enhanced cell survival and adhesion, and the incorporation of negative pressure ventilation aided epithelium survival [136]. Following these two key studies, multiple studies for lung de- and recellularization have been reported in mouse, rat, and pig models [41,101,102,119,137,138,139,140,141,142]. A unique and difficult aspect of lung recellularization is regenerating functional lung microvascular niches. Earlier studies have reported decellularization protocols that maintain lung microvasculature post-decellularization [35,143,144]. However, the functionality of these microvasculature niches remains to be regenerated [145]. Zhou et al.’s porcine lung recellularization study in 2018 reported recellularization using human airway progenitor cells and HUVECs. The authors reported mature endothelial lining in the pulmonary vasculature. Following a 6 day recellularization culture period, porcine transplantation was performed using these bioengineered lungs. The recellularized lungs withstood blood perfusion and demonstrated evidence of gas exchange via blood gas analyses for 1 h post-transplantation [142]. Despite such promising results at a large-scale level, the comparative gas exchange and mechanical compliance was still lower in the recellularized lungs relative to the native lungs. O’Neill and colleagues previously reported a comparative porcine and human lung decellularization study, where both scaffolds could support pulmonary cells. Interestingly, human lung scaffolds had higher metabolic activity and increased stiffness [146]. Platz et al. undertook a novel study for lung transplantation whereby α-gal knock-out pigs were used for de- and recellularization. Multiple cell populations, such as human lung bronchial epithelial cells, bone marrow-derived mesenchymal stromal cells, pulmonary endothelial cells, and lung fibroblasts, were used for recellularization. Interestingly, despite robust cell engraftment, survival, and proliferation, there were no differences between controls and α-gal knock-out decellularized pig lungs [147]. Interestingly, in Song et al.’s study in the rodent model, rodents that received a bioengineered lung transplant had higher oxygenation levels relative to controls 7 days post-transplantation [102]. Recently, Tomiyama and colleagues reported recellularized mouse lungs using a perfusion-based bioreactor with human primary endothelial cells. These bioengineered lungs were transplanted into adult mice and showed pulmonary blood perfusion capability [148]. While small animal models offer great promise for lung recellularization, the recellularization and functionality of recellularized lungs’ scalability to human size still require further effort.

### 4.3. Liver

From the past two decades, several key studies have been reported for liver de- and recellularization. Uygun and colleague’s perfusion-decellularized rat livers, using portal vein perfusion and a modified version of the previously reported heart decellularization study by Ott and colleagues, demonstrated some preliminary recellularization [40,115]. Shupe et al. also reported a rat liver decellularization method and preliminary recellularization findings [149]. Baptista et al. subsequently reported the perfusion de- and recellularization of livers across multiple species such as mice, rat, ferret, and pig [111]. These key studies showed significant advancement for liver bioengineering across multiple animal models and paved the way for future work.

Shupe and colleagues reported the engraftment of WB344 progenitor rat liver cells in their decellularized rat livers following recellularization via the inferior vena cava. Uygun et al. demonstrated an extensive study where the decellularized rat liver matrix could support adult primary hepatocyte engraftment, survival, and metabolic function. Perfusion recellularization studies using microvascular endothelial cells was separately conducted up to 5 days whereby cell engraftment was evident in the vasculature. Transplantation studies also demonstrated a retained vascular network [115]. Baptista and colleagues’ extensive study showed multiple findings. GFP-positive recellularized endothelial cells showed cellular engraftment along the vascular network, human fetal lung cells and HUVECs both show high rates of proliferation and low apoptosis, and metabolic activity was increased in recellularized livers [111].

Following such landmark studies, several studies have optimized decellularization protocols for livers across different animals, while many have also focused on recellularization and re-endothelialization. Liver recellularization has largely reported recellularizations through either a combination of portal vein, hepatic artery, and/or hepatic vein-based seeding. Park and colleagues reported recellularization of porcine livers with porcine iPSC-derived hepatocytes using a continuous perfusion bioreactor system. Their decellularized porcine liver matrix promoted function and maturation of porcine iPSC hepatocytes, and recellularization studies showed attachment of cells to the matrix and distribution of cells throughout the parenchyma by 3–5 days of culture. The heterotopic transplantation of these recellularized porcine livers withstood blood perfusion until 1–2 h of perfusion [150]. Chen and colleagues reported a separate protocol of developing induced hepatocytes from somatic cells and their ability to repopulate decellularized liver matrices [151]. Multiple other studies have also achieved robust recellularization of the endothelium and parenchymal regions in the past few years across different animal models, such as in the mouse [152], rats [120,153,154,155,156,157,158], porcine [152,159,160,161], and cadaveric human livers [121,122]. Many of these studies have shown extents of restored functionality (e.g., measurement of urea, albumin) in recellularized livers. Ko et al. showed that reendothelialized porcine scaffolds were capable of withstanding blood flow and maintaining blood pressure for 24 h post-transplantation [6,160]. Yang et al.’s recellularized mouse liver implantation showed the highest graft survival among previous studies, up to 20–40 days where liver lobule-like tissue formation was observed. The authors also extended their recellularization into pig livers, where recellularized porcine livers were orthotopically transplanted and withstood blood flow up to 18 days [152]. Recently, Shaheen and colleagues demonstrated a recellularized liver, heterotopically transplanted in immunosuppressed pigs, which sustained perfusion up to 15 days [161]. Despite such progress, gaps remain in restoring systemic function to allow bioengineered livers to have biliary excretion and hepatic functions, for example [1].

### 4.4. Pancreas

The pancreas has extensive vascularity, comprising the endocrine and exocrine glands. Therefore, the recellularization of acellular pancreas would require extensive efforts in repopulating the parenchyma, multiple cell types from each of the endocrine and exocrine functions, and the vasculature [6]. Early work from Goh and colleagues reported de- and recellularization of whole mouse pancreas scaffolds, showing the high viability and functional capacity of MIN-6 and AR42J cells after 5 days of culture. In vivo biocompatibility of the decellularized mouse pancreas also revealed good integration with the host [114]. In the same year, separate work by Mirmalek-Sani et al. showed porcine pancreas de- and recellularization where decellularized porcine pancreas was sliced and cultured in 2D culture for recellularization. Static seeding of human amniotic fluid-derived stem cells showed viability and metabolic function 7 days post-seeding [162]. Since then, multiple studies in recent years have reported pancreas de- and recellularization, such as in the mouse [163], rat [112,151,164,165,166,167], porcine [168,169], and human pancreas [170]. Interestingly, Yu and colleagues reported the implantation of a recellularized pancreas in diabetic rats. They used insulinoma and endothelial cells for repopulation whereby, post-transplantation, rats showed euglycemic levels for 7 days [151]. Despite being in a small animal model, such work shows promise for regenerating functional capacities in recellularized whole livers. Guo et al. reported the use of endothelial progenitor cells for recellularization in whole rat decellularized pancreas, where, after 3 days of ex vivo culture and subsequent implantation in rats, evidence of neo-angiogenesis was reported [112]. Katsuki and colleagues reported a multi-step infusion recellularization of decellularized porcine islets via infusion through the pancreatic duct. Repopulated islets showed survival 4 days post-recellularization and insulin secretion levels similar to native levels [169]. Peloso et al. reported the first de- and recellularization of whole human pancreas, where human primary pancreatic endothelial cells showed lining along vessel walls and proliferative capabilities after 6 days of ex vivo culture [170]. For clinical translation, Wan et al.’s study stands out where authors conducted a preliminary trial of recellularizing mouse iPSC-derived pancreatic cells into decellularized rat pancreas. In vivo implantation of decellularized scaffolds showed biocompatibility even after 21 days of implantation. The repopulated scaffolds showed increased insulin levels after 5 days of culture [171].

#### Kidney

Kidney recellularization studies have also progressed significantly in the past two decades. The need and demand for bioengineered kidneys has expanded not only due to circumventing immunosuppression side effects but to also address the shortage of kidney donors. However, kidneys have multiple functions, such as perfusion, filtration, absorption, and the secretion of urine. Kidneys also contained more than 26 types of different cells, adding to their complex composition [13]. As such, bioengineered grafts must also recapitulate these functions to allow clinical translation. Some of the first studies reporting recellularization of whole kidneys had exciting findings [42,172,173,174,175]. Ross and colleagues were the first to report the use of embryonic stem cells for the repopulation of rat kidney scaffolds, showing the cells’ capability to survive and differentiate post-recellularization [174]. Additional work by Ross and colleagues showed success in re-endothelialization of decellularized rat kidneys [175]. Recellularization of porcine kidneys has also shown some promise, where porcine renal cells showed engraftment and capability of forming tubule-like structures by 7 days of culture. These structures were maintained up to 28 days of ex vivo culture [176]. Porcine kidney recellularization has also been attempted, with interesting findings, in additional studies [113,177,178]. Given the complexity of the kidney, promoting recellularization to the perivascular space is also of interest. Some studies have reported success in doing so by perfusing recellularized constructs post-seeding with high pressure to push cells into perivascular spaces [179,180,181]. Others have suggested incorporating a vacuum-system within their bioreactor circuits to promote cell distribution [42,117,173,178,180].

Leuning and colleagues recellularized mouse and human kidneys using iPSC-derived endothelial cells. They repopulated scaffolds via both arterial and venous inlets, reporting enhanced cell coverage [117]. Despite the excitement of using iPSCs, other studies using iPSC cells for repopulated renal scaffolds have shown limitations in achieving proper differentiation [11,172,174,182,183]. Several researchers have reported protocols for deriving iPSC-derived renal progenitor cells that may be useful for recellularization studies [184,185,186]. The recellularization of whole kidneys has, therefore, progressed in a multitude of directions; however, much work remains in achieving the full recellularization of all renal compartments to fully recapitulate and bioengineer whole kidneys.

A summary of key organ recellularization studies have also been summarized in Table 3.

**Table 3:** Summary of key organ (heart, pancreas, lung, kidney, and liver) recellularization studies.

Organ Type, Species	Decellularization Method	Cell Type for Recellularization	Recellularization Seeding (Cell Number and Method)	Duration of Culture	In Vivo Implantation (Y/N)	Analytical Endpoints	Main Conclusions and Findings
*Pancreas*							
Pancreas, rat [112]	Perfusion with PBS 1X 1 h, deionized water 30 m, 0.02% EDTA 30 m, 1% Triton X-100/0.1% ammonium hydroxide until decellularized, PBS 1X wash for 4 h	Bone marrow mononucler cell-derived EPC’s	3 × 10^7^ EPC infused 3 times, 30 m intervals. Static culture for 2 h before machine perfusion (1 mL/min)	3 days Perfusion	Y Recellularizd scaffolds examined 3, 7, 14, and 21 days post-implantation	H&E, CD31, DAPI, DNA quantification, IHC	Decellularized scaffold showed transparent color post-decellularization, loss of DNA content and nuclear content, preservation of ECM content, no graft rejection, few inflammatory cells observed on Day 21 post-implantation, and angiogenesis found after in vivo implantation.
Pancreas, mouse [114]	Retrograde machine perfusion 8 mL/min via hepatic portal vein, 0.5% SDS for 325 m, deionized water 15 m, 1% Triton X-100 15 m, benzonase 15 m, 10% FBS 48 h	MIN-6 cells, AR42J acinar cell	(I) 30 × 10^6^ MIN-6 cells distributed in 3 steps via hepatic portal vein using retrograde gravity perfusion (II) 30 × 10^6^ MIN-6 cells + 30 × 10^6^ AR42J; MIN-6 introduced by vasculature and AR42J by retrograde perfusion via pancreatic duct	5 days Maintained in static culture	Y Decellularized scaffolds implanted and examined 14 days post-implantation	H&E, Masson’s, IHC, TUNEL, Two-photon microscopy, proteomics, DNA, and sGAG quantification, SEM, TEM, atomic force microscopy, qRT-PCR	H&E showed loss of nuclei, DNA quantification show loss of DNA content, IHC shows maintenance of ECM markers, TEM showed intact basement membrane, and SEM showed retained ductal structures. Recellularized cells showed high viability, localization in scaffold after 5 days, and functional capacity (beta cells showed insulin production and acinar cells showed amylase production). In vivo biocompatibility of decellularized scaffold showed active angiogenesis and the presence of mononuclear cells.
Pancreas, porcine [162]	Heparinized PBS wash, 1% Triton X-100/0.1% ammonium hydroxide perfusion for 24 h, PBS rinse for 5 days	hAFSC cells	Unknown number of cells statically seeded on slices of decellularized porcine scaffold for 7 days total	7 days Static seeding in 2D culture	N	H&E, IHC, angiography, viability, ECM quantification, insulin secretion measurements, SEM	H&E showed loss of nuclear content, IHC and quantification of collagen showed maintainenace of ECM components, repopulation showed engraftment and survival of cells up to 3 and 7 days of static culture, and insulin secretion was reported at 3 and 7 days of culture.
Pancreas, human [170]	Pancreatic duct, superior mesenteric artery, splenic artery flushed with heparinized PBS for 1 h, 1% Triton X-100/0.1% ammonium hydroxyide perfusion for 48 h, DNase rinse, saline perfusion for 5 days	hPPEC cells, islets	20 × 10^6^ cells perfused via syringe pump in superior mesenteric artery and splenic artery 200 islets seeded statically on slices of scaffolds	6 days Perfusion 4 days Static culture	N	H&E, angiography, collagen, and DNA quantification, IHC, SEM, CAM, mechanical tests, flow cytometry, Ki67 staining	Clearance of cellular content, intact vascular network observed via imaging, retention of ECM components and loss of DNA content, growth factor retention reported, repopulated cells showed more than 50% proliferation, and endothelial cells confirmed engraftment by CD31 and showed lining along vessel walls.
*Kidney*							
Kidney, porcine [113]	Heparin/PBS 15 m followed by 2 decellularization protocols: (I) 0.5% SDS/DNase via main renal artery for 36 h, PBS wash for 48 h, 500 mL 0.0025% DNase + 10 mM magnesium chloride overnight, PBS rinse 1 h (II) 1% Triton X-100 via main renal artery for 36 h, 0.5% SDS/PBS for 36 h, PBS wash for 72 h	MS1 endothelial cells	300 × 10^6^ cells split into: (I) Static seeding (150 × 10^6^ cells) (II) Perfusion seeding (150 × 10^6^ cells)	3 days Perfusion	N	H&E, DNA quantification, angiography, SEM, blood perfusion in vitro,	H&E and DNA quantification show loss of nuclear content and loss of DNA content, retained morphology, angiograpy shows no extravasation and intact vasculature, both decellularization protocol-treated scaffolds showed cell engraftment on renal arteries and veins, qne whole blood perfusion after recellularization showed multiple sites of extravasation in SDS-treated scaffolds but not Triton X-100 scaffolds. Detachment of recellularized cells was observed after blood perfusion.
Kidney, Mouse and Human [117]	1% SDS perfusion for 12 h, washed with distilled water for 15 m, perfused with 1% Triton X-100 for 30 m, perfused with PBS for 48 h	hgMVECs, hiPSC-derived endothelial cells	Multi-infusion technique; 30 × 10^6^ hgMVECs infused in renal artery followed by 30 × 10^6^ hgMVECs infused in renal vein followed by 10 × 10^6^ hgMVECs infused in renal artery again hiPSC-endothelail cells seeding protocol similar to hgMVECs	13 days Perfusion Monitored at 2 days, 5 days, and 13 days	N	H&E, IHC, ECM maintenance, methylene blue perfusion, proteomics, viability, whole blood perfusion, TEM, SEM	H&E shows cellular clearance, ECM major proteins show retention post-decellularization, preserved microarchitecture reported, multi-infusion recellularization showed enhanced cell coverage and re-endothelialization, and hiPSC-derived endothelial cells show engraftment and survival in scaffolds
Kidney, Rat [172]	Through renal artery, perfusion of 1% SDS for 17 h, perfusion with distilled water	R1 pluripotent mES cells	12 × 10^6^ mES cells infused via artery	24 h or 72 h Perfusion	N	H&E, IHC, microCT, TEM, SEM	Decellularized scaffolds showed retention of structure and inner vasculature tree, and recellularization with mES cells showed uniform cell lining in the vessels and glomeruli at 24 h and 72 h timepoints. High viability rates and low apoptosis rates were found. To determine differentiation of mES cells, loss of Oct4 expression found post-recellularization.
Kidney, Rat [42]	Heparinized PBS via arterial antegrade perfusion for 15 m, 1% SDS for 12 h, deionized water for 15 m, 1% Triton X-100 for 30 min, PBS wash for 96 h	Neonatal kidney cells, HUVECs	50.67 × 10^6^ HUVECs perfusion seeded via arterial inlet, 60.71 × 10^6^ rat neonatal kidney cells seeded via ureter	12 days Perfusion	Y	H&E, IHC, SDS/DNA/collagen/sGAG quantification, TEM, blood/urine chemical analyses, glomeruli quantification	Decellularized kidneys show loss of nuclear content/DNA content and maintenance of ultrastructure. In vitro analyses of de- and recellularized kidneys. Recellularized kidneys had less glomerular filtration than cadaveric kidneys. Increased glomerular filtration and creatinine clearance in decellularized kidneys was reported. Repopulation of endothelial and epithelial cells found after 4 days
*Liver*							
Liver, rat [115]	0.01% SDS perfusion for 24 h, 0.1% SDS for 24 h, 1% SDS for 24 h, wash with distilled water for 15 m, 1% Triton X-100 for 30 m, wash with PBS for 1 h	Hepatocytes	50 or 200 × 10^6^ hepatocytes seeded via multi-step perfusion in 4 steps, 10 min intervals	7–10 days Perfusion	Y 8 h	H&E, IHC, DNA quantification, TUNEL, metabolic activity, viability, corrosion casting	Decellularized scaffolds show maintenance of vascular tree, removal of cellular content, and retention. Recellularization with multi-step seeding showed highest cell seeding efficiency. Some degree of albumin and gene expression reported post-recellularization.
Liver, multiple species (mice/rat/ferret/rabbit/pig) [111]	Distilled water via portal vein 40× the volume of liver, 1% Triton X-100/0.1% Ammonium Hydroxide 50× the volume of liver, distilled water wash Time of decellularization solution per species: mice (1 h), ferret (2 h), rat (3 h), pig (24 h)	MS1 endothelial cells (GFP labeled), hFLCs, HUVECs	100 × 10^6^ MS1 cells injected in vena cava (ferret) followed by machine perfusion 70 × 10^6^ hFLC + 30 × 10^6^ HUVECs cocultured via portal vein (ferret) by perfusion	3 days (MS1) and 7 days (hFLC, HUVECs) Perfusion	Y	H&E, IHC, collagen/sGAG/elastin quantification, SEM, western blot, vascular tree imaging, Ki67, TUNEL	Removal cellular and nuclear content, loss of DNA content, ECM retention, maintained vascular patency, GFP-positive endothelial cells distributed across vascular network, portal vein seeded endothelial cells were located in periportal regions mostly, coculture showed high proliferation and low apoptosis, phenotypic markers found after coculture, and metabolic tests for albumin/urea showed higher concentrations in recellularized scaffolds.
Liver, rat [120]	PBS wash, 0.02% trypsin/0.05% EGTA for 1 h at 37 °C, perfusion of 1% Triton X-100/0.05% EGTA for 18–24 h, perfusion of 0.1% PAA for 2 h, PBS wash	Primary hepatocyte, LSECs	3 × 10^7^ hepatocytes in 30 mL medium via biliary duct. 1.5 × 10^7^ LSECs or HUVECs in 3 mL medium via portal vein	7 days Perfusion	N	H&E, crystal violet staining, IHC, ELISA for albumin, SEM, blood perfusion for 3 h	Decellularization shows loss of nuclear content and maintained vasculature. GFP-positive LSECs found in microvasculature of portal vein post-recellularization, LSEC phenotypic markers show positive expression, CD31 expression also found ubiquitously, metabolic activity higher with coculture setting, and extracorporeal blood perfusion showed maintained blood circulation.
Liver, Rat and Sheep [187]	(I) Perfusion of 0.2 L 1% Triton X-100 for 1 h, wash with distilled water for 1 h, perfusion of 0.05% SDS, wash with water (II) Wash with distilled water for 1 h, perfusion of 0.05% SDS, wash with water Sheep livers used 1% SDS and 5% Triton X-100	GFP-hepatic cells	18 × 10^6^ cells seeded via portal vein	15 days Perfusion	Y 8 weeks	H&E, IHC, biochemical analyses, SEM, mechanical properties testing (tensile strength), MRI, CT angiography	Triton-based decellularization showed promise. Histological analyses showed preservation of microarchitecture of liver scaffolds. No changes in mechanical properties. Maintained ECM components post-decellularization. Recellularization showed GFP-positive cells after 15 days, with hepatocytes engrafted around vessels and in parenchymal spaces.
Liver, human [188]	(I) Perfusion with distilled water, perfusion of 4% Triton-X 100/1% Ammonium hydroxide for 120 min, replaced Triton mixture and re-perfused for 120 min (5 cycles) (II) Perfusion with distilled water, perfusion of 4% Triton X-100/1% ammonium hydroxide for 120 min, perfusion with 1% SDS for 120 min, followed by 120 min Triton perfusion, and 120 min perfusion with SDS	HEPG2 cells	2D culture: 1 × 10^4^ cells/disc 3D culture/bioreactor: 300 × 10^6^ cells injected across 10 intervals	2D culture: 21 days Bioreactor: 5, 8, and 11 days	N	H&E, IHC, SEM, DNA/RNA quantification, GAG and collagen quantification,	Both protocols demonstrated cell clearance and maintenance of tissue structure post-decellularization in both rats and human, reduction in DNA content, increase in collagen and GAG content, and preservation of ECM content reported. In vitro recellularization showed cell presence after 21 days, no abnormal apoptosis or necrosis reported. Bioreactor-based recellularization showed GFP-labeled cells in arteries post-infusion. At day 11, cells were found viable and lining vessel walls.
*Heart*							
Heart, Rat [40]	Heparinized PBS for 15 min, 1% SDS for 12 h perfusion, deionized water perfusion 15 min perfusion, 1% Triton X-100 for 30 min, PBS perfusion for 124 h	Neonatal cardiomyocytes, fibrocytes, endothelial cells, smooth muscle cells, rat aortic endothelial cells	50–75 × 10^6^ combination of cells (Neonatal cardiomyocytes, fibrocytes, endothelial cells, smooth muscle cells) 2 × 10^7^ rat aortic endothelial cells	8–10 days Injection, perfusion 7 days of perfusion re-endothelialization	Y Decellularized scaffolds heterotopically transplanted	H&E, IHC, SEM, mechanical tests, vascular imaging, contractile function testing	SDS-based protocol showed better decellularization results, scaffolds showed loss of nuclear content, loss of DNA content. ECM content was retained post-decellularization. Vascular patency observed post-decellularization. Contracting cell patches observed after 4 days and 9 days in culture during recellularization. Whole heart recellularization showed electric and contractile activity after 8 days, cell engraftment and viability observed, markers of different cell populations also noted after day 8–10 of culture.
Heart, Porcine [39]	Through aorta, hypotonic Type 1 water perfusion for 15 min, PBS perfusion for 15 min, 0.02% trypsin/0.05% EDTA/0.05% sodium azide perfusion for 2 h, 3% Triton X-100/0.05% sodium azie perfusion for 2 h, water perfusion for 5 min, PBS wash for 15 min	Chicken embryonic cardiomyocytes	500,000 cells/cm^2^ seeded on lyophilized decellularized scaffolds	4 days Static seeding	N	H&E, DAPI, IHC, SEM, DNA/sGAG/elastin quantification, ball burst mechanical testing	Decellularization of whole porcine hearts achieved, reduction in DNA content, clearance of nuclear content, preserved ECM components, intact matrix via SEM shown, recellularized scaffolds showed staining of cardiomyocyte sarcomere structure.
Heart, human [130]	Through aorta, anterograde coronary perfusion performed. Perfusion of PBS for 1 h, 1% SDS for 168 h, deionized water for 24 h, 1% Triton X-100 for 24 h, PBS wash for 168 h	BJ-RiPS-derived cardiomyocytes	500 × 10^6^ iPSC-derived cardiomyocytes via intra-myocardial injection into epicardium of left ventricular wall	14 days Perfusion	Y Decellularized scaffolds implanted in rats	H&E, IHC, SDS/DNA/sGAG quantification, collagen/elastin quantification, SEM, TEM, proteomics, biomechanical testing, coronary angiography, microCT, metabolic analyses	Decellularization showed clearance of cellular content, maintained matrix components and ultrastructure of scaffold, 2D recellularization shows evidence of decellularized matrix supporting cells and could be electrically stimulated. Whole heart recellularization showed MHC-positive cells engrafted in left ventricular wall in dense populations, more than 90% viability detected in recellularized cells.
*Lung*							
Lung, Porcine [142]	Perfusion via main pulmonary artery with 0.5% SDS for 30 h, deionized water for 12 h, 1% Triton X-100 for 12 h, PBS wash	Human airway epithelial progenitor cells, HUVECs,	600 × 10^6^ HUVECs seeded through pulmonary artery and pulmonary vein 1 × 10^7^ HUVECs via pulmonary artery and vein + 200 × 10^6^ human airway epithelial cells via bronchus	6 days for both HUVEC only and co-culture of HUVEC + human airway epithelial progenitor cells	Y Recellularized scafflds transplanted in pigs	H&E, DNA/protein quantification, morphometric analyses, proteomics, elastin/sGAG quantification, vascular imaging, mechanical tests, resazurin assay, platelet-rich plasma perfusion, blood-gas analyses	Decellularized scaffolds demonstrated loss of DNA, change in parenchymal area and similar alveolar diameter to natives, ECM preservation found, resazurin assays showed viability of endothelial cells. Co-cultured scaffolds showed proliferative capabilities, lining of epithelial cells along basement membranes, and flattened epithelial cells found in proximity with re-endothelialized capillaries. Transplantation revealed evidence of gas exchange for 1 h.
Lung, rat [135]	Heparinized PBS perfused for 15 min, 0.1% SDS for 120 min, deionized water for 15 min, 1% Triton-X 100 for 10 min	HUVECs, A549, rat fetal lung cells	66.57 × 10^6^ HUVECs seeded via gravity perfusion in pulmonary artery and vein, 91.25 × 10^6^ A549 cells seeded via gravity perfusion through the trachea, 308.57 cells rat fetal lung cells seeded	5–9 days Perfusion	Y Orthotopic transplantation of recellularized scaffolds	H&E, IHC, western blot, morphometric analyses, gas exchange tests, protein concentrations	Low-concentration SDS protocol showed decellularization of scaffolds by histology, ECM factors retained, tissue structure maintained post-decellularization, nuclear remnants detected in trachea only. Re-endothelialized scaffolds showed dispersed cell engraftment of endothelial cells along vascular network after 5 days. Epithelial phenotype observed at 5 days but with longer culture periods, and airway blockage and solid tissue formation was noted. Rat fetal lung cells showed repopulation of scaffold throughout vascular network and no blockage as with A549 cells. Squamous-cell appearing epithelium observed in trachea and airways. Gas exchange evidenced in recellularized scaffolds. Transplantation showed in vivo compatibility for 6 h post-transplantation but recellularized transplants were edematous and required higher pressures.
Lung, rat and human [136]	PBS perfusion for 30 min, CHAPS/EDTA perfusion for 2–3 h, rinse with PBS	For rat: neonatal epithelial cells, lung microvascular cells For human: human endothelial progenitor cell-derived endothelial cells, A549 cells	For rat: 100 × 10^6^ neonatal epithelial cells seeded and perfused after overnight culture. 30 × 10^6^ microvascular cells seeded into vascularature For human: 1 × 10^6^ endothelial or epithelial or co-culture of both seeded onto slices of human lung scaffold	4–8 days Perfusion culture of rat scaffolds 4 days Static culture of human scaffold slices	Y Orthotopic transplantation with recellualrized lungs	H&E, IHC, DNA quantification, collagen/elastin/GAG quantification, western blot, SEM, TEM, microCT, mechanical tests	Nuclear and cellular content cleared post-decellularization, maintained ultrastructure of scaffolds, and ECM proteins preserved after decellularization. Adherence of epithelial cells seen in airway, and microvascular cells showed engraftment in vasculature. Lack of apoptosis and high proliferation observed in recellularized cells. Endothelial cells showed distribution across vasculature, and epithelial cells were found in both large and small airways. Human lung recellularization showed adherence of cells in vasculature and alveoli.

## 5. Discussion

Decellularization and recellularization are essential techniques in TE, with the potential to revolutionize regenerative medicine. While challenges remain, continued advancements in these processes hold promise for creating functional tissue constructs that can significantly improve patient outcomes. Researchers are employing various strategies, such as advanced biomaterials, tissue engineering techniques, and bioreactor systems, to tackle these challenges and advance the field of tissue recellularization towards clinical applications [189].

Ethical concerns regarding the use of animals have led to the widespread development of in vitro recellularization systems. In vitro systems for recellularization can provide fundamental information regarding cellular activities and signaling pathways. In vitro recellularization is an important step that helps ensure the scaffold is properly prepared and functional, thereby increasing the likelihood of successful integration and performance after in vivo transplantation. Cell culture techniques allow cells to grow in vitro under controlled conditions and enable the interaction of cells with a proper ECM, which is also a rich reservoir of growth factors and bioactive molecules that can control cell proliferation and differentiation [190,191]. The evolution, development, and standardization of in vitro techniques will provide vital preclinical indications, enabling informed decisions on whether to advance to animal and human studies [192]. Developing in vitro models is also important for understanding, controlling, and manipulating the immune system [193].

At present, clinical applications of recellularized dECM are still largely in the experimental or early clinical trial stages. However, there are some advancements and applications of dECM in clinical applications. dECM derived from skin has been recellularized and used for many clinical applications. In a 2022 review paper by Kyla Petrie et al., the clinical evidence supporting the use of acellular dermal matrices (ADMs) across various fields, including orthopedic surgery, breast reconstruction, burn and wound care, andrology, oral and maxillofacial surgery, craniofacial surgery, abdominal wall/hernia repair, and otolaryngology, was examined. The early reports on ADMs are promising, but further research is necessary to fully determine their future potential [194]. Tissue engineering of heart valves reseeded with autologous cells has been successfully performed in vitro. Serghei Cebotari and his team reported their first clinical implantation of pulmonary heart valves engineered with autologous endothelial progenitor cells in two pediatric patients, showing good outcomes after 3.5 years of follow-up. These results highlight the importance of creating autologous cardiac valves using tissue engineering methods and underscore their potential benefits for pediatric patients [195]. Additionally, a systematic review and meta-analysis of 17 small studies found that patients with decellularized heart valves implanted in the right ventricular outflow tract had significantly lower reoperation rates compared to those with standard tissue conduits. The authors believe that tissue-engineered heart valves have the potential to revolutionize valve replacement surgery [196]. Further, in 1970, the FDA approved a decellularized bovine carotid vascular graft, and over the past 50 years, this graft has been implanted in more than 500,000 patients for use as hemodialysis access and lower extremity bypass [197]. Using biological matrices that mimic native tissue to varying extents can enhance the biochemical, mechanical, and vascular environment of damaged tissues. This approach can improve cell retention and survival, replicate natural cellular signals, and boost the efficacy of cell-based repair [198]. However, even with the most advanced engineering techniques, extracted and purified proteins cannot capture the complexity of GFs found in native tissue. The ECM produced by stem cells shows great potential for maintaining cell multipotency and inducing a cell’s lineage, and the process of tissue-engineered tissue/organs using dECM has been facilitated by advances in decellularization. However, many decellularization protocols based on SDS and other mechanicals methods can damage the microscopic structure of the ECM and wash away the basic structural proteins [199].

Organ de- and recellularization has undeniably come a long way in the past two decades, with the expansion of this technique across various organs such as the heart, liver, lung, pancreas, and kidney. Researchers have tested various strategies across small and large animal models with multiple cell populations, including iPSC-derived cells.

While many of these advancements bring us closer to using bioengineered organs, some factors remain a challenge in their translation. de Haan et al. discuss how striking a balance between cell removal and ECM preservation leads to challenges for recellularization [11]. Therefore, ongoing efforts are required on both ends of optimizing decellularization protocols to prevent excessive degradation and damage to native microarchitecture while also developing and testing recellularization strategies to achieve adequate cell coverage and function. ECM composition has shown changes in cell behavior and function with disease progression as well as age, which can have a strong influence on recellularization effectivity [11].

Despite many studies on heart, liver, lung, pancreas, and kidney recellularizations reporting re-endothelialization success, we have yet to see evidence for full and long-term re-endothelialization that can be sustained post-implantation. Strategies have been tested to reduce the risk of thrombosis by testing different cell seeding strategies, as reviewed earlier. However, additional considerations and work has also been proposed by testing different ECM coating reagents for re-endothelialization [1]. There is much discourse on the cell sources for recellularization that has yet to be solved. Many of the studies reviewed herein have used cell numbers considerably less than cell numbers found in native organs. For example, while Uygun et al. reported a landmark liver recellularization study in the rat model, they discussed how their cell numbers are comparatively much less (ranging from 5–20%) than that typically found in native livers [1,115]. Understandably, it is difficult to culture and repopulate to such a scale, where some have discussed a possibility of combining both ex vivo culturing and using our own bodies as bioreactors to integrate bioengineered organs fully [13].

As regenerative medicine and TE advance, researchers must still address common challenges to achieve clinical application. The process of repopulating decellularized tissues with new cells faces several challenges that researchers are actively working to overcome. Some of the key challenges include (i) cell sourcing and selection. Identifying appropriate cell sources that can effectively populate and function within the tissue matrix is crucial. These cells must be compatible with the specific tissue type and capable of surviving and thriving in the decellularized scaffold [200,201]. (ii) Cell survival and integration. Ensuring high cell viability and functionality post-implantation is a significant challenge. The cells need to adhere to the scaffold, proliferate, and differentiate appropriately to regenerate functional tissue. Usually, locally administered cells often die before significantly contributing to the healing response due to diffusion limitations of nutrients and oxygen [201]. (iii) Vascularization. Establishing a functional vascular network within the recellularized tissue is critical for nutrient delivery and waste removal. Lack of proper vascularization can lead to limited tissue integration and necrosis. It is well known that scaffold thickness affects cell viability, as most cells in the body are found no more than 100–200 µm from the nearest capillaries, and this is sufficient for the diffusion of oxygen and nutrients [202]. This means that, for the AOS, maintaining the vascular network and venous return during decellularization is crucial, and ensuring continuous, complete reperfusion immediately after transplantation is essential to avoid matrix necrosis. (iv) Immunogenicity and host response. The immune response to the recellularized tissue, including potential rejection or inflammation, must be carefully managed. Challenges with the immunogenicity of dECM scaffolds are categorized as follows: the decellularization protocols used; the effectiveness of antigen removal; the efficiency of washing techniques to eliminate residues; the immunocompatibility of AOS’s sourcing; the degradation rate of dECM to prevent foreign body reactions and chronic immune responses; and the appropriateness of the sterilization method to minimize damage to the ECM [86]. (v) Standardization. Achieving consistency and scalability in the production of decellularized and recellularized tissues/organs for clinical applications is a significant hurdle. Standardized protocols and quality control measures are essential for reproducibility [86,203]. (vi) Functionality. Ensuring that the recellularized tissue maintains its structural integrity, functionality, and phenotype over the long term in vivo remains a challenge. Factors such as the degradation of the scaffold and ongoing cell viability and function need to be addressed [204].

## Abbreviations

TE	Tissue engineering
ECM	Extracellular matrix
AOS	Acellular organ scaffolds
dECM	Decellularized ECM
GFs	Growth factors
GAGs	Glycosaminoglycans
MMPs	Matrix metalloproteinases
TIMPs	Tissue inhibitors of metalloproteinases
LOX	Enzyme lysyl oxidases
TGF-β	Transforming growth factor-β
VEGF	Vascular endothelial growth factor
VEG-A	Vascular endothelial GF-A
EC	Endothelial cells
MMPs	Matrix metalloproteinases
DAMPs	Damage-associated molecular patterns
iPSC	Induced pluripotent stem cells
